# Compensatory selection for roads over natural linear features by wolves in northern Ontario: Implications for caribou conservation

**DOI:** 10.1371/journal.pone.0186525

**Published:** 2017-11-08

**Authors:** Erica J. Newton, Brent R. Patterson, Morgan L. Anderson, Arthur R. Rodgers, Lucas M. Vander Vennen, John M. Fryxell

**Affiliations:** 1 Ontario Ministry of Natural Resources and Forestry, Wildlife Research and Monitoring Section, Trent University, DNA Building, Peterborough, ON, Canada; 2 Department of Integrative Biology, University of Guelph, Guelph, ON, Canada; 3 Ontario Ministry of Natural Resources and Forestry, Centre for Northern Forest Ecosystem Research, Thunder Bay, ON, Canada; 4 Alberta Environment and Parks, Peace River, AB, Canada; University of Tasmania, AUSTRALIA

## Abstract

Woodland caribou (*Rangifer tarandus caribou*) in Ontario are a threatened species that have experienced a substantial retraction of their historic range. Part of their decline has been attributed to increasing densities of anthropogenic linear features such as trails, roads, railways, and hydro lines. These features have been shown to increase the search efficiency and kill rate of wolves. However, it is unclear whether selection for anthropogenic linear features is additive or compensatory to selection for natural (water) linear features which may also be used for travel. We studied the selection of water and anthropogenic linear features by 52 resident wolves (*Canis lupus* x *lycaon*) over four years across three study areas in northern Ontario that varied in degrees of forestry activity and human disturbance. We used Euclidean distance-based resource selection functions (mixed-effects logistic regression) at the seasonal range scale with random coefficients for distance to water linear features, primary/secondary roads/railways, and hydro lines, and tertiary roads to estimate the strength of selection for each linear feature and for several habitat types, while accounting for availability of each feature. Next, we investigated the trade-off between selection for anthropogenic and water linear features. Wolves selected both anthropogenic and water linear features; selection for anthropogenic features was stronger than for water during the rendezvous season. Selection for anthropogenic linear features increased with increasing density of these features on the landscape, while selection for natural linear features declined, indicating compensatory selection of anthropogenic linear features. These results have implications for woodland caribou conservation. Prey encounter rates between wolves and caribou seem to be strongly influenced by increasing linear feature densities. This behavioral mechanism–a compensatory functional response to anthropogenic linear feature density resulting in decreased use of natural travel corridors–has negative consequences for the viability of woodland caribou.

## Introduction

As ecosystems face increasing levels of human-mediated fragmentation and development, interspecies dynamics may also shift [1, 2]. Quantifying the net effects of these changes is important because some species may benefit while others suffer [3–5], potentially creating challenges for conservation [6–9]. In particular, understanding anthropogenic drivers of altered predator-prey relationships is important for making informed decisions regarding species conservation.

The wolf-caribou system of North America’s boreal forest has been an area of particular focus in habitat selection studies [10–12] because woodland caribou (*Rangifer tarandus caribou*) are in decline in North America [13] and globally [14]. Regardless of the proximate agents, anthropogenic factors are often cited as the ultimate driver of these declines [15, 16]. One such mechanism relates to forestry activity that often converts old-growth habitat to early seral forests, which facilitates increases in populations of ungulates such as deer (*Odocoileus virginianus* [17]) and moose (*Alces alces* [18, 19]). Increased ungulate abundance can produce higher densities of predators like wolves, putting caribou at higher risk. Indeed apparent competition (*sensu* [20]) is thought to be the largest cause of declining caribou populations [21, 22].

Wolves (*Canis* sp.) select anthropogenic linear features [23–25], often in relation to their availability [10]. Anthropogenic linear features are associated with faster, more efficient travel [12, 25] and higher prey encounter rates [26–28]; faster moving wolves have been shown to have higher kill rates in Ontario [29]. If an ultimate goal is to characterize habitat selection while hunting, distinguishing between behaviors relating to searching for prey and other behavior (e.g., denning) is important. For coursing predators, "traveling" and "searching for prey" are considered synonymous [30], and traveling can be defined as those times when the predator is not resting or eating [27, 30].

Studies that compare selection of natural to anthropogenic linear features [31, 32] are rare, and the relationship between density of anthropogenic linear features and selection for natural versus anthropogenic linear features by wolves has not yet been described. Selection for linear features is often season-dependent. For example, in the snow-free season–when most caribou mortalities occur in Alberta [33]–industrial linear features were selected for travel by wolves in Alberta, but were not as important during the winter; natural linear features such as waterways were an important feature for wolf movement during all seasons [31]. In Ontario's boreal forest, wolf selection for waterways was more important in an area with lower road density, but seasonal changes in selection were not examined [32].

Our primary focus was to investigate selection by wolves (*Canis lupus* x *lycaon*) for anthropogenic linear features relative to natural linear features such as lake and river edges or frozen streams and waterways using resource selection functions (RSFs [34]). RSFs are frequently used to understand interspecies relationships and habitat use [17, 35, 36]. In a heterogeneous landscape, selection may differ depending on the availability of a given habitat type (i.e., a functional response [10]). The magnitude of variation within this functional response should therefore be proportional to the heterogeneity of the landscape, demanding study at large spatial scales encompassing the full complement of available habitat types.

Previously, the functional response has been studied for *Canis* sp. by comparing selection for anthropogenic linear features among seasons, at different times of day, at different elevations, or to variable levels of prey density [10, 26, 27]. Comparing selection for anthropogenic vs natural linear features is important, because wolves move faster on anthropogenic linear features relative to forested habitats [25, 37], but movement rates on natural linear features in forested habitats have not been quantified. For much of the year velocities on natural linear features are likely less than when on anthropogenic linear features, due to differences in terrain and snow sinking depths [38]. A compensatory relationship between selection for anthropogenic over natural linear features would indicate that wolves switch from using natural to anthropogenic linear features, which may influence prey encounter rates. Industrial development in Ontario is expected to increase [13, 39], and therefore increased knowledge of mechanisms driving wolf habitat selection and interactions with prey in natural and human-modified landscapes is critical to inform conservation efforts for Ontario woodland caribou, which were designated threatened in 2000 [13].

We studied selection for habitat features and the functional response to natural and anthropogenic linear features using telemetry data from 52 wolves in a large geographic area of northern Ontario representing both pristine and human-altered forests. Our objectives were to first establish that wolves in Ontario select linear features and then to determine if selection of anthropogenic linear features was stronger than selection for natural linear features. Anthropogenic linear features such as primary and secondary roads have fewer obstacles, and therefore obstruct movement less, than natural waterways; also, they are generally less tortuous than waterways. Therefore, we predicted that there would be stronger selection and a stronger functional response to anthropogenic linear features compared with natural linear features, particularly in the snow-free season. We predicted that in the winter, selection for all linear features would be similar, because snow could help or hinder travel similarly on roads and waterways. Finally, we determined if use of anthropogenic linear features throughout the landscape was additive or compensatory by comparing availability of anthropogenic linear features with selection for natural and anthropogenic linear features; this could have important implications for management and land use planning. Additive selection in this context would manifest as wolf selection for anthropogenic linear features being unrelated, and in addition to, selection for natural linear features. Compensatory selection of anthropogenic linear features would result in an inverse relationship between selection for anthropogenic linear features and water linear features as anthropogenic linear feature density increases.

## Methods

### Study area

**Fig 1:**
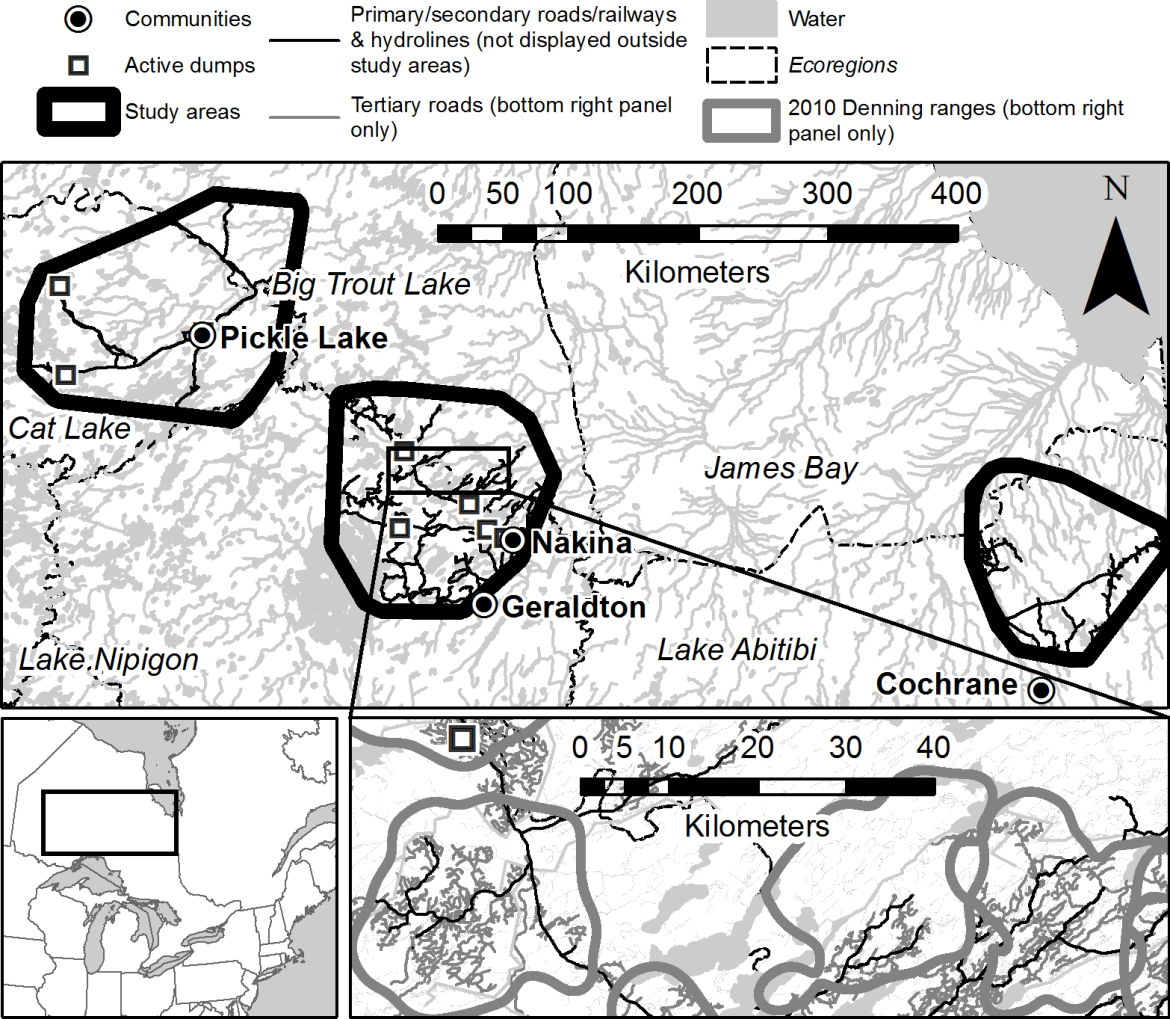
Study areas near Pickle Lake, Nakina, and Cochrane in northern Ontario, Canada, displaying relative size of a sample of seasonal ranges and distribution of linear features within. Study area in top panel and denning ranges in 2010 in bottom right panel. Map data provided by Natural Resources Canada under the federal Open Government Licence–Canada (http://open.canada.ca/en/open-government-licence-canada) and by Ontario Ministry of Natural Resources and Forestry under the Open Government Licence–Ontario (https://www.ontario.ca/page/open-government-licence-ontario).

We studied wolves in three areas totalling 68 000 km^2^ in northern Ontario, ranging in elevation from 200–500 m above sea level and comprising a heterogeneous mosaic of pristine and disturbed forest, provincial parks and protected areas, roads, railways, hydro lines, lakes, rivers, streams and wetlands, 2010–2014 (Fig 1). The Pickle Lake study area (90°34'W, 51°37'N, 28 000 km^2^) was located in eastern Cat Lake Ecoregion and south central Big Trout Lake Ecoregion; the Nakina study area (87°32'W, 50°27'N, 24 000 km^2^) straddles the border between the Lake Nipigon and Big Trout Lake Ecoregions; and the Cochrane study area (80°47'W, 49°58'N, 16 000 km^2^) is located north and east in the Lake Abitibi Ecoregion [40]. Human disturbance was minimal in Pickle Lake, most extensive in Nakina, and intermediate in Cochrane. Common tree species in the study area were black spruce (*Picea mariana*), balsam fir (*Abies balsamea*), white spruce (*P*. *glauca*), tamarack (*Larix laricina*), jack pine (*Pinus banksiana*), white birch (*Betula papyrifera*), and trembling aspen (*Populus tremuloides*); forest stands were a mosaic of coniferous, mixed-woods, and deciduous. January average daily temperatures were -18.6°C and July average daily temperatures were 17.2°C in centrally located Geraldton; mean annual precipitation was 765 mm. In the study area wolves could be hunted or trapped legally 15 Sept–31 March (Fish and Wildlife Conservation Act, 1997). Hunters could take two wolves per season whereas there was no bag limit for trappers trapping on their respective registered trap lines [41]. In the study area, wolves mainly preyed on moose (*Alces alces*), beaver (*Castor canadensis*) and hare (*Lepus americanus*); more rarely, woodland caribou (*Rangifer tarundus*) were preyed upon [42]. Other predators in this system were black bears *(Ursus americanus*), wolverine (*Gulo gulo*) and Canada lynx (*Lynx canadensis*).

### Wolf capture, telemetry, seasons and ranges

Between 31 January 2010 and 30 September 2013, we captured 65 wolves using padded leg-hold traps (#7 EZ grip, Livestock Protection Co., Alpine, Texas) or helicopter net-gunning (Bighorn Helicopters, Cranbrook, BC) and fit wolves with GPS-Argos collars (Lotek 7000MA, 7000SAW, Lotek Inc, Newmarket, Ontario) under Ontario Ministry of Natural Resources animal care permits 10/11/12-218. Mean pack size was larger in Nakina (4.4, range 2–14) than in Pickle Lake (3.7, range 2–7) or Cochrane (3.7, range 2–7). Helicopter-captured wolves were physically restrained for collaring, otherwise, wolves were immobilized using an intramuscular injection of mixed xylazine hydrochloride (2 mg/kg) and Telazol (4m mg/kg); antagonists were either yohimbine (0.15 mg/kg) or atipamezole (0.2 mg/kg). Collars recorded a location every 2.5 or 5 hours depending on the wolf and season; if fix interval changed within a season, we used only locations obtained every 5 hours.

We analyzed wolf selection of linear features during three seasons based on multiple factors: seasonal changes in wolf behaviour and movements observed during our study and other similar studies [43–45], inflection points in tortuosity and velocity of resident wolves in our study area (S1 Fig), and known dates of snow accumulation (e.g., [46]; Environment Canada Climate Normals 1981–2010 data showed that snow cover is > 20 cm per month, November to April). Resulting seasons were denning (1 May–July 31), rendezvous (1 August–31 October), and winter (1 November–30 April). Wolves that had < 50 days of data collection in the first two seasons and < 70 days in winter were excluded from further analysis.

We used R Version 3.0.2 [47] and the Geospatial Modeling Environment Version 0.7.2.1 (Spatial Ecology, 2014) to create 95% seasonal ranges for resident wolves using fixed-kernels and the rule-based *ad hoc* method [48], where increasing proportions of *h*_ref_ were tested until a contiguous range with no internal lacuna formed. Forays lasting < 2 weeks outside established clusters of relocations were removed prior to range creation, as these forays caused the bandwidths calculated using the *ad hoc* method to be unacceptably large. Transient wolves were identified and omitted from all analyses based on a lack of 1) known association with other wolves, and 2) a home range area-curve with no asymptote when 1 random fix per day was examined using the software ABODE V.2-7 [49]. To avoid pseudoreplication, in the rare case when more than one wolf was collared in the same pack during the same season, we analyzed data only from the wolf with the longest active collar duration over that season; data from the other wolf were excluded from the analysis.

### Linear features

We considered roads, hydro lines, railways and water linear features as possible travel corridors for wolves. As vehicular traffic on all anthropogenic linear features was minimal, these features were divided into two categories. The primary/secondary roads/railways and hydro lines category included paved roads, primary and secondary gravel roads driveable with a truck, railways and hydro lines, derived using GIS map layers provided by the Centre for Northern Forest Ecosystem Research (CNFER, Ontario Ministry of Natural Resources and Forestry). These features often overlapped in space or were scarce and it was therefore inappropriate to separate them further. The tertiary roads category included lower-use or grown-over gravel roads and trails (CNFER). Water linear features in the study area were identified using 30 x 30 m resolution data from the Ontario Hydro Network. We removed small water bodies separated by > 100 m from other water and < 500 m in length as they likely did not function as travel corridors for wolves. In all seasons water linear features were defined as waterlines and shorelines; in the winter season virtual lines connecting hydrologic inflow and outflow through centres of lakes and rivers also functioned as linear features to account for wolves crossing large frozen waterbodies. Water lines with very small drainage areas that likely did not represent wolf travel corridors were removed: we used the Flow Accumulation and Stream Grid layers of the Ontario Integrated Hydrology Data (Spatial Data Infrastructure Unit, Ontario Ministry of Natural Resources) and ArcGIS 10.1 to prune the stream network where drainage area was less than 150 cells (i.e. < 0.14 km^2^); 94.7% of the cells representing water lines were retained.

### Habitat

Habitat covariates included in RSF models were derived from the Far North Land Cover map products Version 1.3.1 [50]. The original landcover classes were reduced to eight classes to increase accuracy [51] and reduce number of model parameters. The reduced classes were lowland (freshwater marsh, thicket swamp, treed peatland, open fen, treed fen, open bog, treed bog, and deciduous swamp, 37% of the total area), sparse/barren, (sparse treed, bedrock, 3%), deciduous/mixed forest (deciduous treed and mixed treed, 8%), old disturbance (≤ year 2000 natural disturbance; e.g., burn, pest or disease, unknown disturbance, and unknown disturbance with age unknown, 6%), recent disturbance (> year 2000 natural disturbance, 2%), old cuts (≤ year 2000 forest cuts, 3%) and recent cuts (> year 2000 forest cuts, 4%). Lowland and new disturbance are used proportionally less than their availability by wolves in this study area [52, 53], whereas old disturbance is selected, both in Ontario and elsewhere in North America [10, 32, 52]. Wolves also typically select areas preferred by moose and also areas with higher moose density, such as deciduous/mixed forests and cuts; coniferous forests may be selected or avoided by moose, depending on the season and density of cover [10, 32, 52–54]. To account for wolves selecting areas preferred by moose, we included both old and new cuts, as the age of a cut block influences moose selection [55]. We included sparse/barren areas in the models as these areas may be selected differentially in summer and winter by wolves [10], and because wolves may use large rocks for denning and rendezvous sites [56], which could affect selection coefficients. The remainder of the study area was made up of water linear features (6%) and anthropogenic linear features (2%); other classes with minimal coverage were not included in the analysis. Habitat density, including density of linear features, was calculated as the number of habitat feature pixels (1 pixel = 30 x 30 m) divided by the total number of pixels in each seasonal range. Disturbance and cut classes reflect the year the majority of telemetry data were collected (2010); less than 0.2% of the study area was converted to recent cuts between 2010 and 2012, so we used 2010 data for all years. The area had little topographical relief and we did not include elevation in our models.

### Statistical analyses

We conducted a Euclidean distance-based [57, 58] RSF at the home-range scale, also known as Johnson’s [59] third order selection to compare locations used by wolves to locations available within their seasonal ranges. This approach measures the distance from each location to the nearest habitat feature of each type. For brevity, we use the term "selection" for a habitat feature to indicate that wolves selected areas closer to that feature than what was randomly available on the landscape. We chose a generalized linear mixed-model approach over a movement-based model of availability because it is more appropriate for resource selection studies at larger spatial scales [60]. We wished to analyze data from traveling wolves only. To reduce spatial autocorrelation and to filter out most non-traveling locations when wolves were resting or eating at kill, den, rendezvous and bedding sites, we removed locations at known feeding sites (within 1 km of dumps) and excluded locations with velocity to previous or next location < 50 m/hr; this filter was more conservative than that used to define traveling in Whittington et al. [27], but less conservative than that used in Dickie et al. [25], especially considering our 5-hour fix interval.

To ensure that available points accurately represented available habitat [57], we randomly selected two seasonal ranges from each study area (six total) and overlaid between 0.2–10 random points/km^2^ throughout the ranges; random points were chosen from a uniform distribution. We followed Obbard et al. [61] to determine the random point density at which the distance to features stabilized at the representative seasonal ranges. The median distance to features stabilized at 4 points/km^2^; therefore, we selected this point density for further analysis. The mean ratio of used to available locations was 1:14.3 (S1 Table). To ensure that used and available points were sampled from the same area, we removed any wolf locations that occurred outside of their respective 95% seasonal range.

Territorial behaviour generally prevents resident wolves from accessing features inside another wolf’s range [62–65]. All used and available locations were within the wolf's seasonal range. However, because selection could reflect knowledge of features occurring just outside the wolf's seasonal range, we measured distance to features within an area representing the seasonal range buffered by 7 km. This buffer size represented the 75th percentile of the maximum distance resident wolves were found outside their 95% seasonal range boundary before filtering extra-territorial locations.

From each used or available location, we measured distance in km to the nearest linear feature and habitat type. These measurements were standardized before inclusion in the RSF models by subtracting the season and feature-wise median [27] and dividing by 1.5 x the interquartile range. This is the non-parametric equivalent to subtracting the mean and dividing by two standard deviations, a practice recommended to improve model performance and facilitate comparison among variables that differ in magnitude [66, 67].

Some habitat classes or linear features did not occur in all seasonal ranges. We compared two approaches to deal with this issue, and ran the full analysis, described below, for each method. The "Maximum Distance" method assigns the measured distances between observed or random locations and features missing from a given territory the value corresponding to the greatest distance between a wolf location and the feature within the overall dataset [43]. The "Functional Availability" method, proposed here, includes an interaction term (1 or 0) in both fixed and random effects to indicate whether each habitat variable was available (or unavailable, respectively) to the wolf. Because we found the Functional Availability method to be analytically superior, with similar results, we present results only for that method in the main body of the text. S1 File includes further details and results for the Maximum Distance method for purposes of comparison. Some features that were technically available according to the above criteria were very scarce within individual territories; accordingly, we only considered a feature “available” if it occurred within the seasonal range and the density was greater than 1% for land cover features and greater than 0.1% for anthropogenic linear features.

We estimated a mixed-effects logistic regression model for each season using a hierarchical model selection approach e.g., [68–70]. Although we could have calculated individual models for each wolf, year, and season [60], using mixed effects models allowed us to account for differences in sample size and availability of each habitat type when calculating population and individual-level selection coefficients, while also accounting for correlation due to some individuals being measured over several years [10, 11, 71]. First, to test for multicollinearity we investigated Pearson correlation coefficients (*r*) and variance inflation factors (VIF) using SAS PROC REG for each season using the global fixed effects structure and no random effects. We removed or modified independent variables such that in any given model *r* < 0.70 and VIF < 3 for all factors [70, 72]. Next, we tested a set of models (listed in Table 1), each containing all possible fixed-effects covariates (global fixed effects), but with varying random effects: we tested among models with no random effects, models with random intercepts for wolf and year, and models with random intercepts and random coefficients (slopes) for water linear features, primary/secondary roads/railways and hydro lines, and tertiary roads and combinations therein (Table 1). We selected the optimal random effects structure using the Akaike information criterion (AIC). We also included results for the Bayesian information criterion (BIC), because AIC may select overly complex models when sample sizes are high [73, 74]. Random intercepts account for unbalanced sample sizes across animals and years; random coefficients model individual-level patterns in selection by allowing the slope of the logistic regression to vary for each feature (when specified), individual and year [11, 71, 75, 76]. Finally, we retained the random effects structure in the most highly supported model, then tested among a number of fixed-effects structures (Table 2), again including results for both BIC and AIC. To account for any remaining autocorrelation among telemetry locations we specified an empirical (sandwich) estimator. We used five-point adaptive Gauss-Hermite quadrature [11, 77] to obtain more accurate parameter estimates, and data were processed by subjects (between-within degrees of freedom) using SAS PROC GLIMMIX (Version 9.3; SAS Institute, Cary, NC, USA). Restricted maximum likelihood estimates are not well defined for binomial GLMMs and therefore we used maximum likelihood in both steps of hierarchical model selection.

**Table 1:** Candidate model set with varying random effects structures and global fixed effects structure* for three seasons, describing selection of landscape variables by wolves in northern Ontario, Canada, 2010–2014.

Random effects	AIC^†^	BIC^†^	ROC^†^	ΔAIC^†^	*w*_*i*_^†^
**Denning**					
**water + primary/secondary roads/railways & hydro lines × A + tertiary roads × A | Wolf ID / Year**	53686.13	53713.55	0.7392	0.00	1
**water + primary/secondary roads/railways & hydro lines × A | Wolf ID / Year**	53914.60	53940.30	0.7338	228.47	0
**water + tertiary roads × A | Wolf ID / Year**	53939.92	53965.63	0.7338	253.79	0
**primary/secondary roads/railways & hydro lines × A + tertiary roads × A | Wolf ID / Year**	54183.25	54208.95	0.7288	497.12	0
**water | Wolf ID / Year**	54223.88	54247.87	0.7278	537.75	0
**primary/secondary roads/railways & hydro lines × A | Wolf ID / Year**	54418.82	54442.81	0.7230	732.69	0
**tertiary roads × A | Wolf ID / Year**	54433.13	54457.12	0.7225	747.00	0
**Intercept = 1 | Wolf ID / Year**	54732.43	54754.71	0.7144	1046.30	0
**no random effects**	56133.68	56250.25	0.6843	2447.55	0
**Rendezvous**					
**water + anthropogenic linear features × A | Wolf ID / Year**	54339.11	54360.73	0.7831	0.00	1
**water | Wolf ID / Year**	55159.32	55179.28	0.7662	820.21	0
**anthropogenic linear features × A | wolf ID / year**	55309.57	55329.53	0.7666	970.46	0
**1 | wolf ID / year**	56150.30	56168.60	0.7485	1811.19	0
**no random effects**	57688.79	57785.92	0.7266	3349.68	0
**Winter**					
**water + anthropogenic linear features × A | wolf ID / year**	72257.00	72280.48	0.7001	0.00	1
**anthropogenic linear features × A | wolf ID / year**	72711.42	72733.10	0.6890	454.42	0
**water | wolf ID / year**	72881.98	72903.66	0.6837	624.98	0
**1 | wolf ID / year**	73347.99	73367.86	0.6728	1090.99	0
**no random effects**	74875.56	74975.90	0.6430	2618.56	0

"A" is a dummy variable coded 1 or 0 depending on whether the habitat class was available or unavailable, respectively, to the wolf.

*Fixed effects for the denning model were coniferous forest + sparse/barren + deciduous/mixed forest + lowland + recent disturbance × A + old disturbance + old cuts × A + recent cuts × A + water + primary/secondary roads/railways & hydro lines × A + tertiary roads × A. For the rendezvous and winter seasons, fixed effects were the same, except recent cuts were removed, and anthropogenic linear features replaced the variables tertiary roads and primary/secondary roads/railways & hydro lines.

^†^Column names are AIC = Aikake information criterion, BIC = Bayesian information criterion, AUC = area under the ROC curve, ΔAIC = delta AIC, *w*_*i*_ = AIC weight.

**Table 2:** Candidate model set with varying fixed effects structures* and top-model selected random effects structures for three seasons, describing selection of landscape variables by wolves in northern Ontario, Canada, 2010–2014.

Random effects	Fixed effects	AIC^†^	BIC^†^	ROC^†^	ΔAIC^†^	*w*_*i*_^†^
**Denning**						
**water + primary/secondary roads/railways & hydro lines×A + tertiary roads×A |** **wolf ID / year**	global (habitat + cuts + disturbance + linear features)	53686.13	53713.55	0.7392	0	1
	habitat + disturbance + linear features	53725.56	53749.55	0.7384	39.43	0
	habitat + cuts + linear features	53749.93	53773.92	0.7383	63.80	0
	habitat + linear features	53778.79	53799.35	0.7378	92.66	0
	cuts + disturbance + linear features	53800.57	53821.13	0.7378	114.44	0
	disturbance + linear features	53833.84	53850.98	0.7371	147.71	0
	cuts + linear features	53862.65	53879.79	0.7369	176.52	0
**Rendezvous**						
**water + anthropogenic linear features×A |** **wolf ID / year**	global (habitat + cuts + disturbance + linear features)	54339.11	54360.73	0.7831	0	1
	habitat + disturbance + linear features	54391.56	54411.52	0.7823	52.45	0
	habitat + cuts + linear features	54419.22	54437.52	0.7816	80.11	0
	habitat + linear features	54464.83	54481.47	0.7809	125.72	0
	cuts + disturbance + linear features	54808.32	54823.29	0.7771	469.21	0
	disturbance + linear features	54847.09	54860.40	0.7764	507.98	0
	cuts + linear features	54866.59	54878.23	0.7757	527.48	0
**Winter**						
**water + anthropogenic linear features×A |** **wolf ID / year**	global (habitat + cuts + disturbance + linear features)	72257.00	72280.48	0.7001	0	0.852
	habitat + disturbance + linear features	72260.50	72282.18	0.7001	3.50	0.148
	habitat + cuts + linear features	72292.83	72312.71	0.6993	35.83	0
	habitat + linear features	72295.32	72313.39	0.6993	38.32	0
	cuts + disturbance + linear features	72686.74	72703.00	0.6921	429.74	0
	disturbance + linear features	72698.08	72712.54	0.6921	441.08	0
	cuts + linear features	72712.45	72725.10	0.6915	455.45	0

"A" is a dummy variable coded 1 or 0 depending on whether the habitat class was available or unavailable, respectively, to the wolf.

*The fixed effects categories are as follows: habitat (coniferous forest + sparse/barren + deciduous/mixed forest + lowland), cuts (denning = old cuts × A + recent cuts × A; winter and rendezvous = old cuts × A), disturbance (recent disturbance × A + old disturbance), and linear features (denning = water + primary/secondary roads/railways & hydro lines × A + tertiary roads × A; winter and rendezvous = water + all anthropogenic linear features × A).

^†^Column names are AIC = Aikake information criterion, BIC = Bayesian information criterion, AUC = area under the ROC curve, ΔAIC = delta AIC, *w*_*i*_ = AIC weight.

To determine if there was a functional response to linear features, we assessed the relationship between density of a linear feature within each wolf’s territory and the selection coefficient (from the RSF models, outlined above) for linear feature types using linear mixed models with the function lmer in the “lmerTest” package in R [78] with wolf ID as a random effect to control for multiple years of measurement of the same wolf. We examined functional responses to both natural and anthropogenic linear feature density. Because our overall objective was to better understand how anthropogenic linear features may alter wolf movements, we were interested in determining whether the relationship between selection for anthropogenic linear features and natural linear features was additive or compensatory. We expected that if wolves used anthropogenic linear features independently of water linear features, indicating additive use, the selection for both feature categories would be unrelated or change in concert as density of anthropogenic linear features increased. An inverse relationship between water and anthropogenic linear features as anthropogenic linear feature density increased would indicate compensatory use. We assessed functional response relationships when the marginal R^2^_GLMM_, defined as the variance explained by the fixed effects [79], was > 0.1 [11] and when relationships were significantly different than zero (*P* < 0.05).

Data are available from the Dryad Digital Repository: [*http://dx.doi.org/10.5061/dryad.t5800*].

## Results

After removing data from wolves that either had insufficient data or were in the same pack as another included animal, rarifying locations to a common fix interval per season, removing locations that were associated with very small extraterritorial movements and removing locations that were associated with kills and bed sites, 23.1% of the location data remained, resulting in 26 387 locations available for analysis. A breakdown of the number of locations per wolf and season is provided in S1 Table. Of the 52 wolves that were suitable for analysis, median active collar duration was 183 days (range 59–780 days), for a total of 35, 32, and 35 wolves studied for denning, rendezvous, and winter seasons, respectively. Some wolves were studied over multiple years, so sample sizes per year and season were 41, 38, and 44, respectively. Wolves in the same pack generally have similar selection coefficients so a larger number of wolves in disparate packs increases effective sample size [25, 26, 31, 80]. To reduce collinearity in the model sets during the rendezvous and winter seasons, we combined the variables primary/secondary roads/railways and hydro lines and tertiary roads into a new variable, anthropogenic linear features, and removed the variable recent cuts. These variables were not as highly correlated in the denning seasonal ranges (due to the location of these ranges throughout the study area); because we were interested in differences between types of anthropogenic linear features, we did not combine them for denning models. After these changes, for all fixed effects and seasons, VIF was < 1.92 and *r* was ≤ 0.662.

Certain features were not available to some wolves throughout each season. Two to 15 wolves could not access anthropogenic linear features (anthropogenic linear features, tertiary roads, or primary/secondary roads/railways and hydro lines), depending on the season (S2 Table). Seven wolves did not have access to recent disturbance in their seasonal ranges, and 7 to 17 wolves did not have recent or old cuts in their seasonal ranges (S2 Table).

During the first step of model selection (choosing an appropriate random effects structure while holding fixed effects constant), in all three seasons, the top model was always the model with the most complex random effects structure (Table 1). The minimum AIC, BIC, and maximum ROC were consistent within the model set and all three criteria selected the same top models. During the second step of model selection (assessing best fixed effects after choosing the appropriate random effects structure), the most complex model was ranked first by the model selection procedure. In addition, the AIC, BIC, and ROC consistently selected the same top model—the model with global fixed effects structure: habitat variables, cuts, disturbance, and linear features (Table 2). Both IC methods favoured models that contained several non-significant predictor variables, even when we tried other combinations (not presented here) with only fixed effects that were significant in the top models.

**Fig 2:**
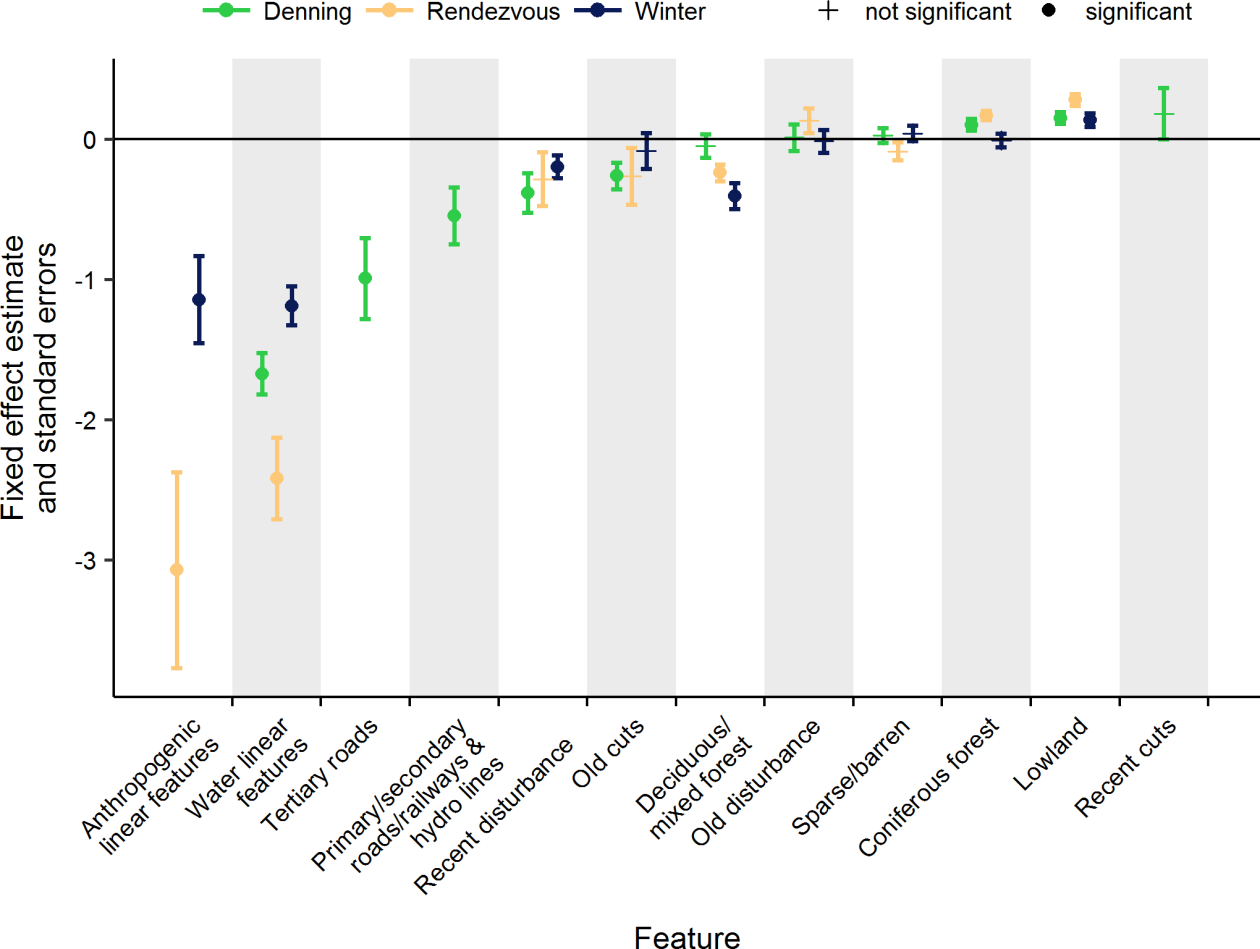
Parameter estimates and standard errors for fixed effects in the top selected models using the Functional Availability method, describing selection of landscape variables by wolves in northern Ontario, Canada, 2010–2014. Symbols indicate significance for *α* = 0.05. Negative values indicate selection.

**Fig 3:**
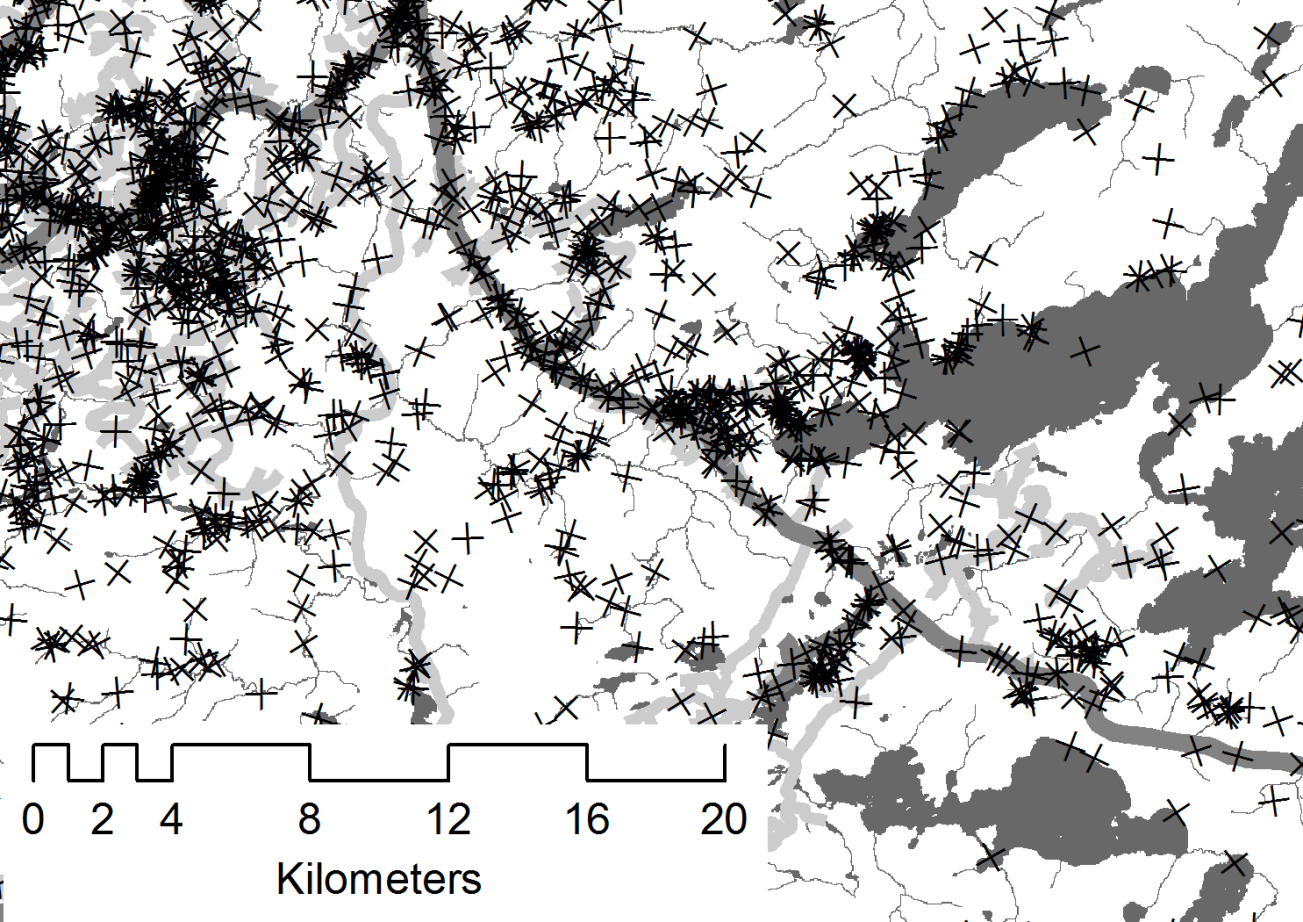
Filtered GPS fixes (crosses) of 5 wolves in the Nakina study area, 2010–2014. Dark grey shading and thin lines are streams, rivers, and lakes; thick dark lines are primary roads, and thick light lines are tertiary roads.

**Fig 4:**
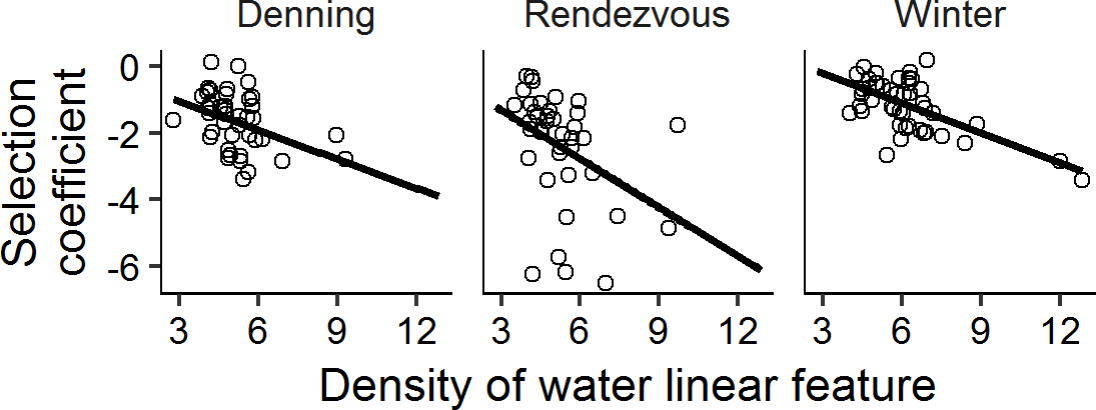
Functional response relationships between density of water linear features and selection for water linear features for wolves in northern Ontario, Canada, 2010–2014. All slopes were significantly different than zero (P < 0.027 for all relationships); R^2^_GLMM(m)_ for each relationship was > 0.15. Negative selection coefficients indicate selection.

Selection in a Euclidean distance analysis indicates that individuals selected areas that on average were closer to the feature of interest than would have been the case had they used the landscape randomly. Among the final top models for each season, the fixed effects most strongly selected for in each season were anthropogenic linear features and water (Fig 2). Indeed, selection for all linear features was readily apparent in the telemetry data used for analysis (e.g., Fig 3). Wolves weakly selected against lowland in all three seasons. Weak selection for other features varied by season: in the denning season, wolves selected recent disturbances, old cuts, and did not select coniferous forests. In the rendezvous season, wolves selected deciduous mixed forests but selected against coniferous forests. In winter, wolves selected recent disturbance and deciduous mixed forests. In the rendezvous season, anthropogenic features were selected more strongly than water, and selection for all linear features was stronger in the rendezvous season than in denning and winter. Covariances for random effects in the top models for each season indicated that selection was most variable in the rendezvous season and for anthropogenic linear features in the winter, but were comparable within the denning season for both types of anthropogenic linear features (Table 3, Fig 4).

**Table 3:** Covariance and standard errors for random effects in the top selected models for each season describing selection of landscape variables by wolves in northern Ontario, Canada, 2010–2014.

Feature	Denning	Rendezvous	Winter
Intercept	0.227 ± 0.048	0.706 ± 0.140	0.184 ± 0.038
anthropogenic linear features × availability	-	14.141 ± 4.929	3.016 ± 1.668
tertiary roads × availability	1.195 ± 0.764	-	-
primary/secondary roads/railways & hydro lines × availability	1.218 ± 0.513	-	-
water	0.797 ± 0.166	2.853 ± 0.860	0.684 ± 0.209

**Fig 5:**
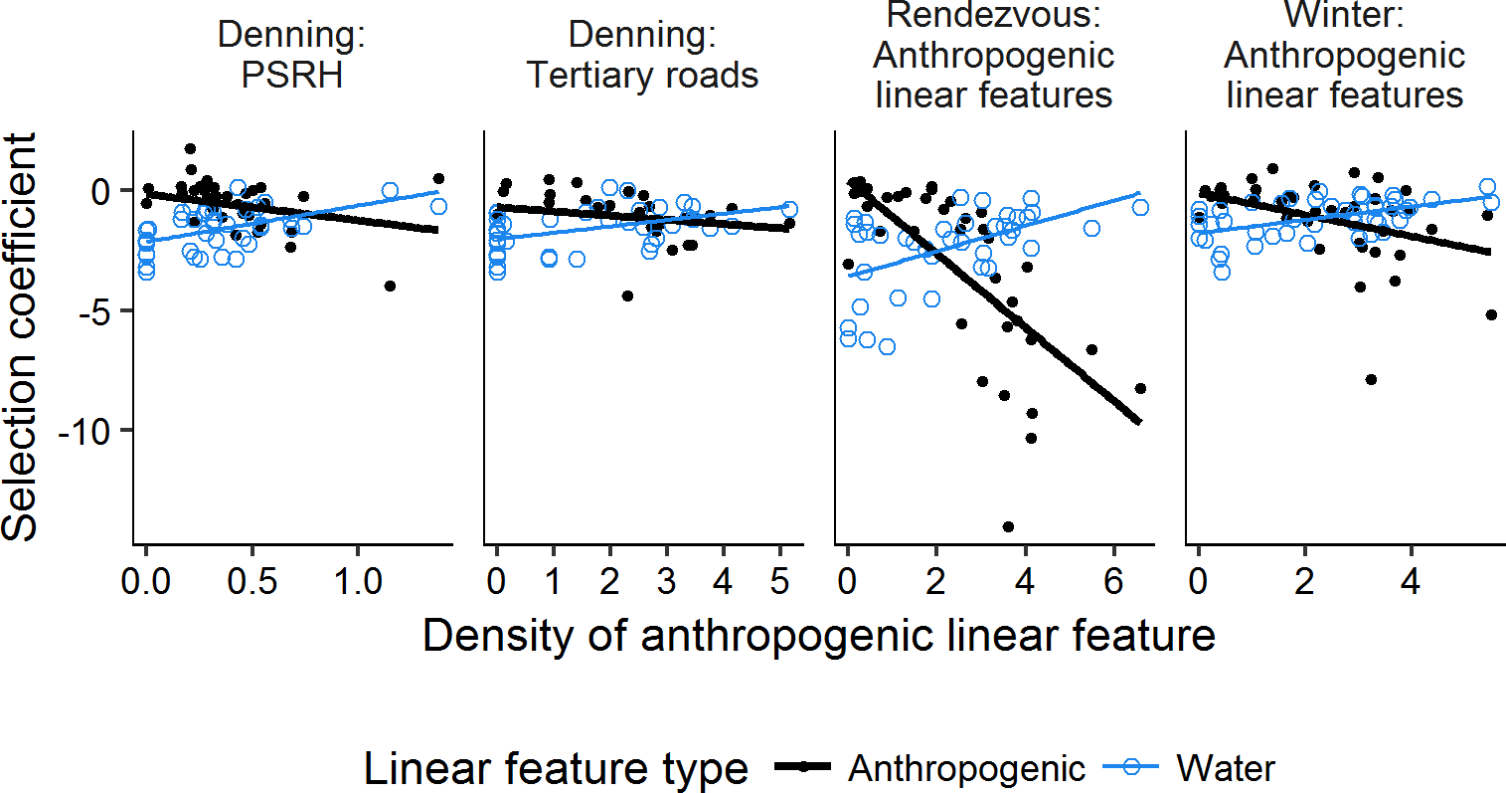
Functional response relationships between density of anthropogenic linear features and selection for anthropogenic and natural linear features by wolves in northern Ontario, Canada, 2010–2014. All slopes were significantly different than zero (P < 0.037 for all relationships); R^2^_GLMM(m)_ for each relationship was > 0.11. Negative selection coefficients indicate selection. PSRH = Primary/secondary roads/railways & hydrolines.

We found evidence for a significant functional response to water linear features in all seasons (Fig 4). As density of natural linear features increased, selection also increased. The functional response to water linear features was strongest during the rendezvous season (Fig 4). In all seasons of the year, as anthropogenic linear feature density increased, wolf selection for these features also increased (all slopes were significantly below zero) while selection for natural linear features decreased accordingly (all slopes were significantly greater than zero; selection is indicated by negative selection coefficients, Fig 5). Very few wolves exhibited positive or neutral selection coefficients for anthropogenic linear features (i.e., it was rare that wolves used those features proportionally less compared to their availability). When weakly positive coefficients did occur it was generally at low to moderate linear feature densities. The strongest functional response to anthropogenic linear features occurred during the rendezvous season. The most marked decrease in selection for water with increased density of primary/secondary roads/railways and hydro lines occurred during the denning season (Fig 5, note scale of x-axis).

## Discussion

We studied selection by resident wolves for anthropogenic and natural linear features in a heterogeneous landscape in northern Ontario. We show evidence supporting a functional response to the density of water and anthropogenic linear features during all seasons of the year. Wolves selected areas closer to water when it was more available on the landscape, but we found an inverse relationship between selection for natural and anthropogenic linear features. Specifically, as density of anthropogenic linear features increased, selection for those features increased and selection for natural linear features decreased. While we did not investigate total time spent on each linear feature type, this evidence strongly suggests at least partially compensatory use of anthropogenic vs natural linear features, as additive use would manifest as no relationship between selection for natural vs anthropogenic linear features and density of anthropogenic linear features on the landscape.

Apparent competition causing declines of caribou across their range is a broadly studied phenomenon, and increased predation of caribou due to increases in wolf abundance or increased ability of wolves to permeate the landscape have been implicated as causal mechanisms [6, 21, 27, 81]. Our study represents an important mechanistic puzzle-piece not previously demonstrated: wolves with greater access to roads and other human-made corridors select natural linear features such as lake and river edges and streams less. Our research further emphasizes the strong selection for anthropogenic linear features by wolves and the potential importance of these features in mediating wolf movements, and by extension potentially influencing encounter rates with prey.

We note that the global fixed effects structure was selected as the top model, even though non-significant factors were present. We suspect this effect could be due to false precision due to high sample sizes of “available” points compared to “used” points. However, we believe our model was biologically relevant and that using a large number of “available” points allowed us to more accurately model habitat within each home range. Further, while using rescaled variables in our models was useful in determining relative importance of features and enabled model convergence, it did not facilitate direct comparison between selection for anthropogenic to natural features.

Wolves selected anthropogenic and water linear features throughout the year, but the strength of selection varied seasonally. Compared to selection for anthropogenic linear features, the odds of wolves selecting water linear features were higher in the denning season, similar in winter, and lower in the rendezvous season. We suggest that these differences reflect a shifting balance in optimal foraging strategies as accessibility of prey items and permeability of the landscape changes throughout the year. During the denning season wolves may use natural features as corridors more frequently because they select denning areas close to water [56] and likely make shorter hunting forays. Riparian habitats are also frequently visited in summer because beaver are an important food source [82]. Further, in the snow-free season large lakes and major rivers may act as at least partial barriers. For example, migrating caribou are deflected by large open water bodies and often follow the water’s edge to reach their destination [83]. Therefore, selection may be in part a side effect of large water bodies acting as barriers in the ice-free season. Whereas Dickie et al. [25] found that wolves selected long, straight linear features over more narrow and sinuous features, we found that during the denning season wolves selected tertiary roads (which in our study were short, narrow, and sinuous) more often than primary/secondary roads/railways and hydro lines.

Wolves often cover large distances in winter [84–86] and frozen waterways are an important travel corridor [37, 87]. In this season travel on all linear features could be facilitated by compacted, hard-crusted snow or impeded by deep, soft snow [25, 85, 88]. Both plowed and unplowed anthropogenic linear features were associated with lower sinking depths than natural areas in Alberta, making them energetically more favourable for wolf travel during the winter, but plowed roads were associated with much lower sinking depths than any other habitat type [38]. In our study area, most tertiary roads and many secondary roads were not maintained during winter, and therefore likely represented similar sinking depths to rivers, but were likely lower (more favourable) than other natural areas [38].

The rendezvous season possibly represents the most favourable energetic balance for wolves using anthropogenic linear features. By nature, anthropogenic linear features likely facilitate faster travel than water linear features during the snow-free season, but to our knowledge, movement rates on both types of features have not been compared. During the rendezvous season, wolves generally switch from smaller prey such as beaver to larger prey [82, 89] and may use anthropogenic linear features preferentially to facilitate faster travel and increased efficiency before roads become snowed in and when rivers and lakes are still open. Moose also prefer early seral stage forests associated with recent cuts and tertiary roads in northern Ontario, and because wolves may select habitat preferred by prey [32, 90], traveling on these features could increase hunting success. We also note that during the winter and rendezvous seasons, recent cuts had to be excluded from the candidate models because they were too highly correlated with tertiary roads. During denning season, models indicated that recent cuts were not used. Even though we followed accepted standards on when to exclude related variables, we believe that the parameter estimate indicating less use of recent cuts in the denning season is an artifact of multicollinearity.

In general, throughout the year wolves selected recent disturbances, old cuts, and deciduous mixed forests and did not use coniferous forests and lowlands. Although we filtered the data to remove many locations associated with bed or kill sites, some selection may have still represented general use and not features used for traveling. Overall, selection was likely related to the tendency for wolves to select areas where prey forage resources are high [32, 90, 91], and to not use areas with low prey density or that hinder travel. For example, moose—the main ungulate prey of wolves in northern Ontario [42, 91]—prefer early successional, disturbed, and deciduous mixed forests [45, 53, 54, 92]–habitat types that were selected by wolves in this study. Caribou are known to “space away” from predators and choose habitat not frequented by wolves. Evidence suggests caribou select coniferous forests and lowlands and do not use disturbed areas [93–95].

This study has important implications for studies of wolf movement rates on anthropogenic linear features and "other" habitat, especially if all "non-linear feature" habitat is grouped together (e.g. [25, 37]). If wolves use waterways as travel corridors, their speed on these features may be increased compared to in wetlands or forests. Therefore, studies investigating speed of travel "off" linear features should include natural linear features as a separate habitat class. Further, we studied only resident wolves, but because transient wolves have different movement patterns and may encounter caribou more often than resident wolves [27], their space use and behaviour should be incorporated in future work to understand the full complement of effects linear features may have on wolf-caribou interactions. Our methods would have been unable to estimate habitat availability for transients, but approaches based on step selection functions [96, 97] or using Brownian bridge models (e.g. [98]) would overcome this hurdle.

Wolves displayed a functional response to anthropogenic linear features during all seasons. Areas containing multiple or wider features suitable for travel are used more often than areas with greater landscape resistance [99]. Therefore, wolves living in ranges containing a dense network of anthropogenic linear features may select these features disproportionately, as it allows them to move more efficiently across the landscape. It is possible that at low densities, anthropogenic linear features are novel on the landscape and therefore relatively unused (indicated by positive coefficients), but that at increasing densities anthropogenic linear features become more familiar and useful as travel routes. Hebblewhite and Merrill [10] found that selection was related more to the amount of human activity than to anthropogenic linear feature density. However, in our study area human activity overall was low, and density of anthropogenic linear features was likely more important.

In landscapes with a low level of logging activity, road density strongly influenced wolf space use [24, 45]. Houle et al. [45] found no functional response to road density in the denning season, and in contrast to our findings, they showed that in the rendezvous and winter seasons wolves did not use roads when road density was high [45]. However, sample size in their study was low compared to ours and human activity was likely much higher than in our study area. In our study, only a few wolves had individual selection coefficients that indicated less use of anthropogenic linear features compared to their availability, and all but one of these occurred at low to moderate anthropogenic linear feature densities (Fig 5). The only wolf that showed some evidence of not using primary/secondary roads/railways and hydro lines at high anthropogenic linear feature density had a denning territory that surrounded a small town.

A functional response to density of water linear features was evident in each season in our study area. Water was fairly ubiquitous in all study areas compared to the density of anthropogenic linear features. A functional response to these features in the snow-free seasons likely reflects increased use of riparian habitats for hunting and greater mobility along connected waterways and lake edges. In winter, frozen waterways are excellent travel corridors for wolves and represent a significant improvement in mobility over other landscape types [38].

The variation in selection coefficients for anthropogenic and natural linear features was markedly larger in the rendezvous season compared to denning or winter. This pattern may be related to division of labour within the pack: wolves have different roles within the pack during the rendezvous season, with mothers generally staying closer to pups, and other pack members becoming more active to compensate [100]. Wolf packs tend to be more cohesive during winter with the pack functioning more as a single unit [85, 101]. This likely accounts for the lower variability in selection coefficients documented during winter.

### Conclusions

Throughout the year, decreased selection for water linear features was correlated with greater density and selection for anthropogenic linear features. We demonstrated that selection of anthropogenic linear features is at least partially compensatory–depending on the density of these features, wolves may switch from using natural corridors to anthropogenic features. Previously, increases in wolf velocity across the landscape, related to increased density and use of roads, were linked to higher kill rates of moose in our study areas [28]. Future studies estimating the impact of anthropogenic linear features on wolf-prey dynamics should not overlook the compensatory relationship between use of these features and natural linear features. Quantifying the effects of increasing density of different types of linear feature classes on predator-prey relationships is complicated because use is not necessarily additive. Future research should quantify wolf movement rates on anthropogenic versus natural linear features and attempt to predict the net effects of varying degrees of anthropogenic disturbance on the rates of encounter between wolves and their prey. A further consideration is that if wolves switch from using natural to anthropogenic linear features, the relative use of different prey species may change not only because of increased velocity and success in these areas [25–28], but because natural corridors (waterways) where prey such as beaver are more likely to be encountered are used less. Managers should consider the potential impact of new linear features on the landscape that are located far from natural travel routes such as rivers and lakes as these may facilitate wolf use of areas that would otherwise be avoided. Specifically, we recommend that any efforts to rehabilitate or add new anthropogenic linear features should consider the arrangement of the water network. Viewing the road and water network as a whole will paint a more comprehensive picture of wolf travel routes and potential implications for woodland caribou.

## Supporting information

S1 Table Mean and range of number of used or available locations for each wolf-year-season combination. (PDF)

S2 Table Summary of how many wolf-season-year combinations did not have a feature available within a seasonal range. (PDF)

S1 Fig Season delineation, tortuosity and velocity of resident adult wolves in northern Ontario, Canada, 2010–2014. Season limits are shown with a thin grey line.
(PDF)

S1 File Results for the Maximum Distance method. Comparison of the Maximum Distance vs Functional Availability methods.
(PDF)
