# Supplementary material for: Compensatory selection for roads over natural linear features by wolves in northern Ontario: Implications for caribou conservation
Source: PLoS One. 2017 Nov 8;12(11):e0186525. doi: 10.1371/journal.pone.0186525 (PMC5695599; doi:10.1371/journal.pone.0186525)
Supplement: S1 Table — (PDF) [file pone.0186525.s001.pdf]

S1 Table. Mean and range of number of used or available locations for each wolf-year-season combination.

	Denning	Rendezvous	Winter
Used locations	195 (68-335)	222 (75-331)	227 (83-505)
Available locations	2787 (1230-6781)	2912 (970 – 6894)	3514 (1140 – 8516)
