# Supplementary material for: Compensatory selection for roads over natural linear features by wolves in northern Ontario: Implications for caribou conservation
Source: PLoS One. 2017 Nov 8;12(11):e0186525. doi: 10.1371/journal.pone.0186525 (PMC5695599; doi:10.1371/journal.pone.0186525)
Supplement: S2 Table — (PDF) [file pone.0186525.s002.pdf]

S2 Table. Summary of how many wolf-season-year combinations did not have a feature available within a seasonal range.

**Season**	**Feature**	**Available**	**Not Available**	**Percent Unavailable (%)**
**Denning**	anthropogenic linear features	33	8	20
	old cuts	24	17	41
	recent cuts	26	15	37
	recent disturbance	34	7	17
	primary/secondary roads/railways & hydro lines	32	9	22
	tertiary roads	26	15	37
**Rendezvous**	anthropogenic linear features	37	2	5
	old cuts	29	10	26
	recent cuts	32	7	18
	recent disturbance	32	7	18
	primary/secondary roads/railways & hydro lines	37	2	5
	tertiary roads	31	8	21
**Winter**	anthropogenic linear features	41	4	9
	old cuts	35	10	22
	recent cuts	38	7	16
	recent disturbance	38	7	16
	primary/secondary roads/railways & hydro lines	41	4	9
	tertiary roads	36	9	20
