# Supplementary material for: Compensatory selection for roads over natural linear features by wolves in northern Ontario: Implications for caribou conservation
Source: PLoS One. 2017 Nov 8;12(11):e0186525. doi: 10.1371/journal.pone.0186525 (PMC5695599; doi:10.1371/journal.pone.0186525)
Supplement: S1 Fig — Season limits are shown with a thin grey line. (PDF) [file pone.0186525.s003.pdf]

To aid in delineating seasons for wolves in Ontario, we calculated velocity (km/hr) and tortuosity (fractal dimension; [1, 2]) of resident wolves using only data from days when locations were taken every 15 minutes (S1 Fig). Season limits are shown with a thin grey line.

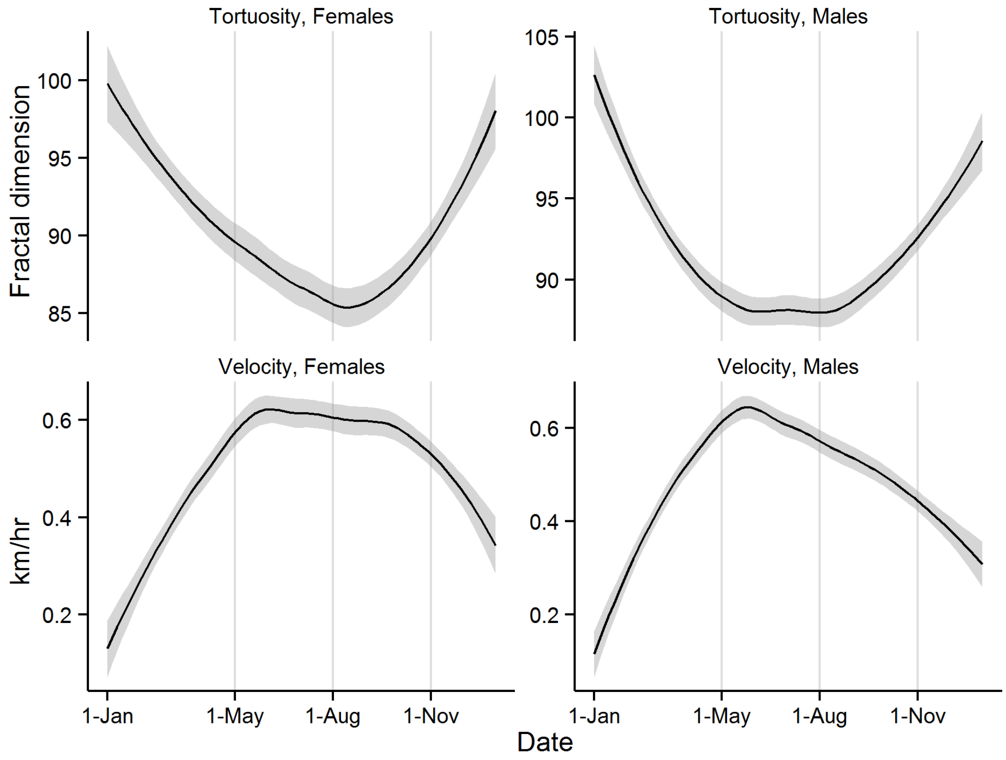

S1 Fig. Seasonal delineation, tortuosity and velocity of resident adult wolves in northern Ontario, Canada, 2010-2014.

References

1. Bascompte J, Vilà C (1997) Fractals and search paths in mammals. Landsc Ecol 12:213–221
2. Mills K, Patterson B, Murray D (2006) Effects of variable sampling frequencies on GPS transmitter efficiency and estimated wolf home range size and movement distance. Wildl Soc Bull 34:1463–1469
