# Supplementary material for: Compensatory selection for roads over natural linear features by wolves in northern Ontario: Implications for caribou conservation
Source: PLoS One. 2017 Nov 8;12(11):e0186525. doi: 10.1371/journal.pone.0186525 (PMC5695599; doi:10.1371/journal.pone.0186525)
Supplement: S1 File — Comparison of the Maximum Distance vs Functional Availability methods. (PDF) [file pone.0186525.s004.pdf]

S1 File

All figures and tables referenced in this supplemental file are within this document.

Results for the "Maximum Distance" method

In all seasons the fixed effects old and recent cuts were removed due to collinearity, and we used the variable anthropogenic linear features (combining primary/secondary roads/railways & hydro lines, and tertiary roads) in winter. After these changes VIFs were < 2.03 and r values were ≤ 0.653.

When fixed effects were held constant in all three seasons, the top model was not always the model with most complex random effects structure, and in one case, the maximum ROC was not consistent with the minimum AIC and BIC (S3 Table). In the denning season, the random effect of tertiary roads was dropped and in the rendezvous season, the random effect of primary/secondary roads/railways & hydro lines was dropped. When assessing the fixed effects structure after selecting the appropriate random effects structure, the global model was selected (S4 Table). Both IC methods favoured models that contained several non-significant predictor variables.

Among the final top models for each season, the fixed effects most strongly selected for in each season were anthropogenic linear features and water (S5 Table). Fixed effects estimates for the Maximum Distance method were comparable to the Functional Availability method for landscape variables, but differed for linear features (compare S5 Table to S6 Table). Selection for water was as strong or stronger than selection for anthropogenic linear features in all three seasons, including rendezvous. With the exception of tertiary roads in the denning season and primary/secondary roads/railways & hydro lines in the rendezvous season, selection for

anthropogenic linear features was stronger than all other habitat variables in the models (S6 Table).

With respect to random effect parameter estimates, results for the Maximum Distance method were similar to the Functional Availability method (S7 Table, S2 Fig).

Wolf functional response to water was similar using the Maximum Distance method compared to the Functional Availability method (S3 Fig). Increased density of roads, trails, hydro lines and railways were associated with decreased selection for water linear features (S4 Fig), similar to the Functional Availability method.

Summary and conclusions

The Functional Availability and Maximum Distance methods differed in the treatment of animals without access to certain linear features. We used a hierarchical model selection approach wherein the optimal random effects structure was chosen first and fixed effects structure second. In the Maximum Distance method, the random effects for some anthropogenic linear features were dropped in the first step of model selection. When random effects were present, selection coefficients compared between both methods were quite similar. However, when a wolf could not access a feature, selection for that feature would appear as neutral selection in the Maximum Distance model, contributing to decreased variance within random effects overall. We believe this is the reason that tertiary roads were dropped in model selection from the Maximum Distance denning models. Further, we believe that PSRH was dropped as a random effect in the Maximum Distance method during the rendezvous season because tertiary roads and PSRH were correlated – not strongly enough to combine them into a single variable as we did in the Functional Availability method, but enough that perhaps the variability among

wolves could be more parsimoniously attributed to selection for one linear feature rather than both.

Overall, the Functional Availability method allowed us to include more variables in the candidate model set because including interactions between landscape variables and their availability reduced collinearity. While we cannot directly compare parameter estimates between the Functional Availability method and the Maximum Distance method because of differing random and fixed effect habitat variables being selected in the top models, parameter estimates indicating avoidance of habitat types that were not available to all wolves tended to be lower in the Functional Availability method, a trend that matched our predictions. In general, we recommend against using the Maximum Distance method because key predictors could be lost in model selection if they are not available to most animals.

S3 Table. Candidate model set with varying random effects structures and global fixed effects structure for three seasons using the Maximum Distance method, describing selection of landscape variables by wolves in northern Ontario, Canada, 2010-2014. Column names are AIC = Aikake's information criterion, BIC = Bayesian information criterion, AUC = area under the ROC curve, ΔAIC = delta AIC, w_i = AIC weight.

Fixed	Random	AIC	BIC	ROC	ΔAIC	w_i
**Denning**						
coniferous forest + sparse/barren + Water + deciduous/mixed forest + lowland + recent disturbance + old disturbance + tertiary roads + primary/secondary roads/railways & hydro lines	Water + primary/secondary roads/railways & hydro lines | wolf ID / year	54010.59	54032.87	0.7331	0.00	0.649
	tertiary roads + primary/secondary roads/railways & hydro lines + Water | wolf ID / year	54011.82	54034.10	0.7384	1.23	0.351
	Water + tertiary roads | wolf ID / year	54067.20	54089.48	0.7334	56.61	0
	Water | wolf ID / year	54299.77	54320.33	0.7272	289.18	0
	primary/secondary roads/railways & hydro lines | wolf ID / year	54514.45	54535.02	0.7222	503.86	0
	tertiary roads + primary/secondary roads/railways & hydro lines | wolf ID / year	54520.14	54540.70	0.7280	509.55	0
	tertiary roads | wolf ID / year	54566.70	54587.27	0.7218	556.11	0
	Intercept = 1 | wolf ID / year	54811.87	54830.72	0.7134	801.28	0
	no random effects	56258.54	56355.68	0.6830	2247.95	0
**Rendezvous**						
coniferous forest + sparse/barren + Water + deciduous/mixed forest + lowland + recent disturbance + old disturbance + tertiary roads + primary/secondary roads/railways & hydro lines	Water + tertiary roads | wolf ID / year	54615.40	54637.03	0.7777	0.00	1
	Water + primary/secondary roads/railways & hydro lines | wolf ID / year	54760.73	54782.36	0.7767	145.33	0
	tertiary roads + primary/secondary roads/railways & hydro lines + Water | wolf ID / year	54767.91	54789.53	0.7764	152.51	0
	Water | wolf ID / year	55237.25	55257.21	0.7663	621.85	0
	tertiary roads + primary/secondary roads/railways & hydro lines | wolf ID / year	55334.46	55356.08	0.7671	719.06	0
	tertiary roads | wolf ID / year	55592.40	55612.36	0.7608	977.00	0
	primary/secondary roads/railways & hydro lines | wolf ID / year	55760.56	55780.52	0.7596	1145.16	0
	1 | wolf ID / year	56254.45	56272.75	0.7483	1639.05	0

	no random effects	58218.04	58315.17	0.7221	3602.64	0
**Winter**						
coniferous forest + sparse/barren + Water + deciduous/mixed forest + lowland + recent disturbance + old disturbance + anthropogenic linear features	Water + anthropogenic linear features | wolf ID / year	72291.50	72313.18	0.7001	0.00	1
	anthropogenic linear features | wolf ID / year	72747.42	72767.29	0.6888	455.92	0
	Water | wolf ID / year	72896.24	72916.12	0.6837	604.74	0

S4 Table. Candidate model set with varying fixed effects structures and top-model selected random effects structures for three seasons using the Maximum Distance method, describing selection of landscape variables by wolves in northern Ontario, Canada, 2010-2014. Column names are AIC = Aikake's information criterion, BIC = Bayesian information criterion, AUC = area under the ROC curve, ΔAIC = delta AIC, w_i = AIC weight.

	**Random**	**Fixed**	**AIC**	**BIC**	**ROC**	**ΔAIC**	**w_i**
**Denning**	Water + primary/secondary roads/railways & hydro lines | wolf ID / year	Global (Habitat + disturbance + linear features)	54010.59	54032.87	0.7331	0.00	1
		Habitat + linear features	54048.80	54067.65	0.7322	38.21	0
		Disturbance + linear features	54167.88	54183.30	0.7306	157.29	0
**Rendezvous**	Water + tertiary roads | wolf ID / year	Global (Habitat + disturbance + linear features)	54615.40	54637.03	0.7777	0.00	1
		Habitat + linear features	54675.53	54693.83	0.7763	60.13	0
		Disturbance + linear features	-	did not converge		-	
**Winter**	Water + anthropogenic linear features | wolf ID / year	Global (Habitat + disturbance + linear features)	72291.50	72313.18	0.7001	0.00	1
		Habitat + linear features	72320.48	72338.55	0.6992	28.98	0
		Disturbance + linear features	72727.69	72742.14	0.6921	436.19	0

S5 Table. Parameter estimates and standard errors for fixed effects in the top selected models for each season using the Functional Availability method describing selection of landscape variables by wolves in northern Ontario, Canada, 2010-2014. Negative values indicate selection.

**Feature**	**Denning**	**Rendezvous**	**Winter**
Intercept	-2.884 ± 0.080*	-3.172 ± 0.143*	-2.746 ± 0.066*
anthropogenic linear features×A	-	-3.073 ± 0.699*	-1.145 ± 0.310*
Water	-1.674 ± 0.147*	-2.420 ± 0.290*	-1.189 ± 0.138*
tertiary roads×A	-0.994 ± 0.289*	-	-
primary/secondary roads/railways & hydro lines×A	-0.546 ± 0.202*	-	-
recent disturbance×A	-0.382 ± 0.141*	-0.286 ± 0.192	-0.198 ± 0.081*
old cuts×A	-0.262 ± 0.095*	-0.265 ± 0.204	-0.085 ± 0.129
deciduous/mixed forest	-0.049 ± 0.084	-0.240 ± 0.061*	-0.406 ± 0.093*
old disturbance×A	0.011 ± 0.094	0.132 ± 0.089	-0.016 ± 0.083
sparse/barren	0.026 ± 0.054	-0.087 ± 0.063	0.041 ± 0.055
coniferous forest	0.102 ± 0.042*	0.168 ± 0.033*	-0.009 ± 0.050
lowland	0.151 ± 0.042*	0.280 ± 0.043*	0.134 ± 0.049*
recent cuts×A	0.181 ± 0.183	-	-

S6 Table. Parameter estimates and standard errors for fixed effects in the top selected models for each season using the Maximum Distance method describing selection of landscape variables by wolves in northern Ontario, Canada, 2010-2014. Negative values indicate selection.

Feature	Denning	Rendezvous	Winter
Intercept	-2.397 ± 0.250*	-2.716 ± 0.188*	-2.649 ± 0.085*
anthropogenic linear features	-	-	-1.121 ± 0.319*
Water	-1.653 ± 0.146*	-2.569 ± 0.324*	-1.163 ± 0.133*
tertiary roads	-0.110 ± 0.080	-1.523 ± 0.347*	-
primary/secondary roads/railways & hydro lines	-0.546 ± 0.189*	-0.248 ± 0.133	-
recent disturbance	-0.277 ± 0.136*	-0.260 ± 0.169	-0.160 ± 0.071*
deciduous/mixed forest	-0.040 ± 0.082	-0.237 ± 0.059*	-0.408 ± 0.094*
old disturbance	-0.006 ± 0.092	0.089 ± 0.094	-0.028 ± 0.082
sparse/barren	0.030 ± 0.056	-0.087 ± 0.067	0.043 ± 0.054
coniferous forest	0.122 ± 0.041*	0.160 ± 0.034*	-0.012 ± 0.049
lowland	0.168 ± 0.040*	0.272 ± 0.042*	0.135 ± 0.049*

S7 Table. Covariance and standard errors for random effects in the top selected models for each season using the Maximum Distance method describing selection of landscape variables by wolves in northern Ontario, Canada, 2010-2014.

**Feature**	**Denning**	**Rendezvous**	**Winter**
Intercept	0.416 ± 0.275	0.677 ± 0.231	0.202 ± 0.055
anthropogenic linear features	-	-	2.565 ± 1.349
tertiary roads	-	3.788 ± 1.311	-
primary/secondary roads/railways & hydro lines	1.026 ± 0.627	-	-
Water	0.807 ± 0.170	2.501 ± 0.670	0.688 ± 0.211

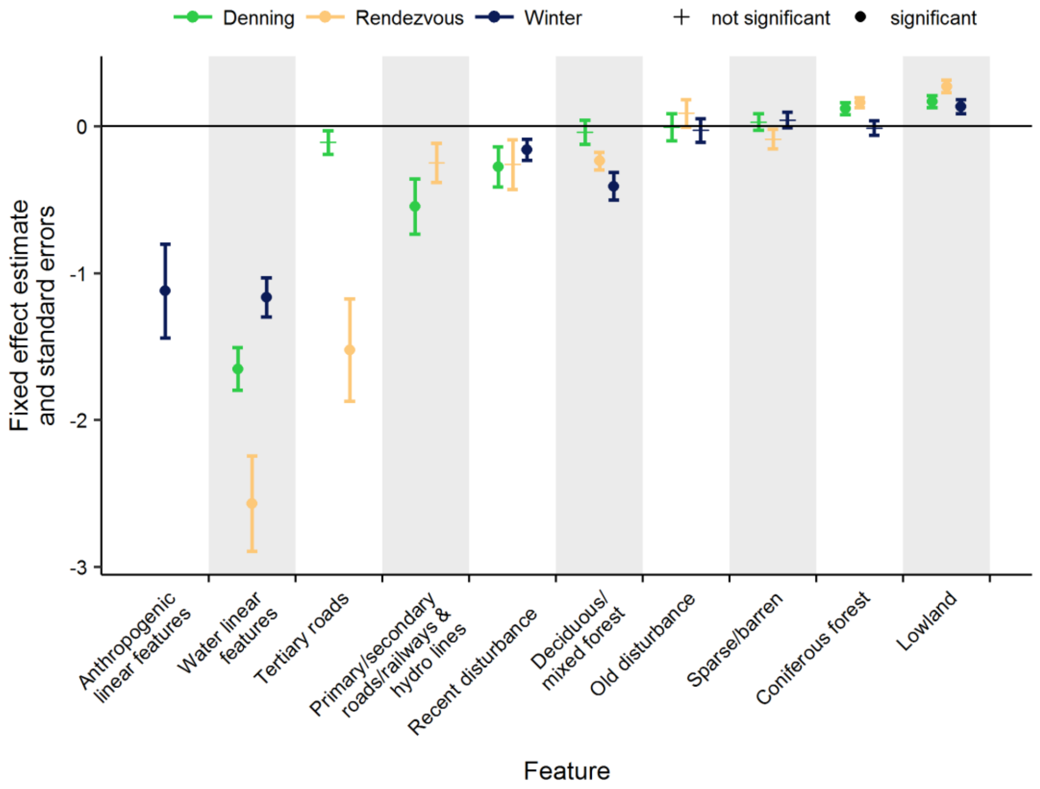

S2 Fig. Parameter estimates and standard errors for fixed effects in the top selected models using the Maximum Distance method describing selection of landscape variables by wolves in northern Ontario, Canada, 2010-2014. Symbols indicate significance for $\alpha = 0.05$. Negative values indicate selection.

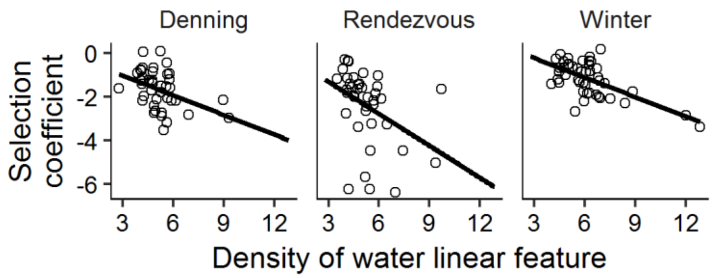

S3 Fig. Functional response relationships between density of water linear features and selection for water linear features for wolves in northern Ontario, Canada, 2010-2014 using the Maximum Distance method. All slopes were significantly different than zero ($P < 0.024$ for all relationships); $R^2_{GLMM(m)}$ for each relationship was > 0.14. Negative selection coefficients indicate selection.

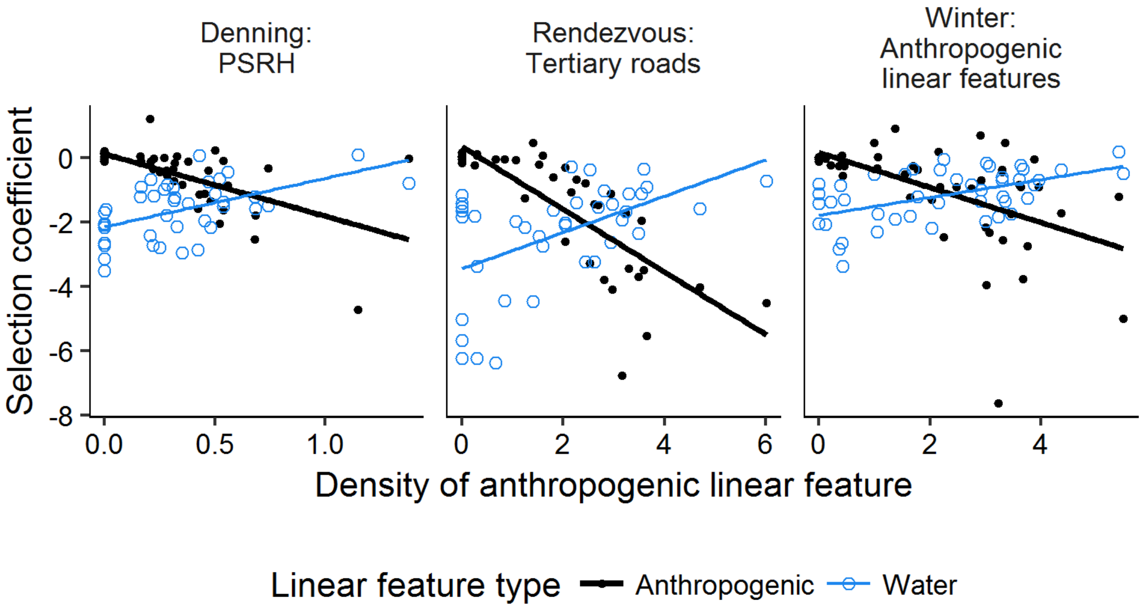

S4 Fig. Functional response relationships between density of anthropogenic linear features and selection for anthropogenic and natural linear features by wolves in northern Ontario, Canada, 2010-2014 using the Maximum Distance method. All slopes were significantly different than zero ($P < 0.001$ for all relationships); $R^2_{GLMM(m)}$ for each relationship was > 0.23. Negative selection coefficients indicate selection. PSRH = Primary/secondary roads/railways & hydrolines.
